# Advances and Techniques in Subcuticular Suturing for Abdominal Wall Closure: A Comprehensive Review

**DOI:** 10.7759/cureus.65069

**Published:** 2024-07-21

**Authors:** Maulik Maheshwari, Imran Ali Khan

**Affiliations:** 1 General Surgery, Jawaharlal Nehru Medical College, Datta Meghe Institute of Higher Education and Research, Wardha, IND

**Keywords:** clinical outcomes, cosmetic outcomes, barbed sutures, surgical techniques, abdominal wall closure, subcuticular suturing

## Abstract

Subcuticular suturing has emerged as a prominent technique for abdominal wall closure, offering notable benefits in cosmetic outcomes, infection reduction, and patient satisfaction. This comprehensive review delves into the evolution and current state of subcuticular suturing, examining its principles, techniques, and advancements. Traditional methods like continuous and interrupted suturing are compared with modern innovations like barbed sutures and knotless techniques. Clinical outcomes, including healing efficacy, complication rates, and cost-effectiveness, are analyzed to highlight the technique's advantages. The review also explores specific applications in various surgical specialities, presenting case studies and clinical trials to substantiate its effectiveness. Despite certain challenges and limitations, the future of subcuticular suturing appears promising with ongoing research and technological advancements. This review aims to thoroughly understand subcuticular suturing, emphasizing its significance in improving surgical outcomes and patient care in abdominal wall closure.

## Introduction and background

Closing the abdominal wall is a critical step in many surgical procedures, impacting both the immediate- and long-term outcomes of the surgery. Proper closure techniques are essential for preventing complications such as infections, hernias, and dehiscence, which can significantly affect patient recovery and overall health [1]. The integrity of the abdominal wall also plays a crucial role in maintaining the structural and functional stability of the body, as it helps contain and protect internal organs while supporting bodily movements and posture [2]. Abdominal wall closure involves meticulously approximating various tissue layers, including the peritoneum, muscle, fascia, and skin. The closure method must ensure adequate tensile strength to withstand intra-abdominal pressures and promote optimal healing. Innovations and refinements in suturing techniques have continually evolved to improve the efficacy and safety of these procedures [3].

Suturing techniques for abdominal wall closure have advanced significantly over the years, encompassing a variety of methods tailored to different surgical needs and patient conditions. Traditional techniques such as simple interrupted and continuous suturing have been widely used for their simplicity and reliability. However, these methods often require meticulous knot-tying and may be time-consuming [4]. Subcuticular suturing, in particular, has gained popularity due to its ability to provide excellent cosmetic results with minimal scarring. This technique involves placing sutures in the dermal layer beneath the skin surface to approximate the wound edges without visible external sutures. It can be performed using absorbable or nonabsorbable sutures, and various materials and needle types are employed to achieve optimal results [5]. Other advanced techniques, including barbed sutures, knotless suturing, and tissue adhesives, have been introduced to enhance the efficiency and effectiveness of abdominal wall closure. These innovations reduce operative time, minimize tissue trauma, and improve wound healing outcomes [6].

This comprehensive review aims to provide an in-depth analysis of the advances and techniques in subcuticular suturing for abdominal wall closure. By examining the historical development, current practices, and recent innovations in this field, the review seeks to offer valuable insights for clinicians and researchers. It will evaluate the clinical outcomes, efficacy, and challenges associated with subcuticular suturing and its applications in various surgical specialties. The review will also highlight emerging trends and future directions in abdominal wall closure techniques, emphasizing the importance of continued research and development. This review aspires to improve surgical practices and patient care in abdominal wall closure by synthesizing existing knowledge and presenting the latest findings.

## Review

Subcuticular suturing techniques

Basic Principles of Subcuticular Suturing

**Figure 1:**
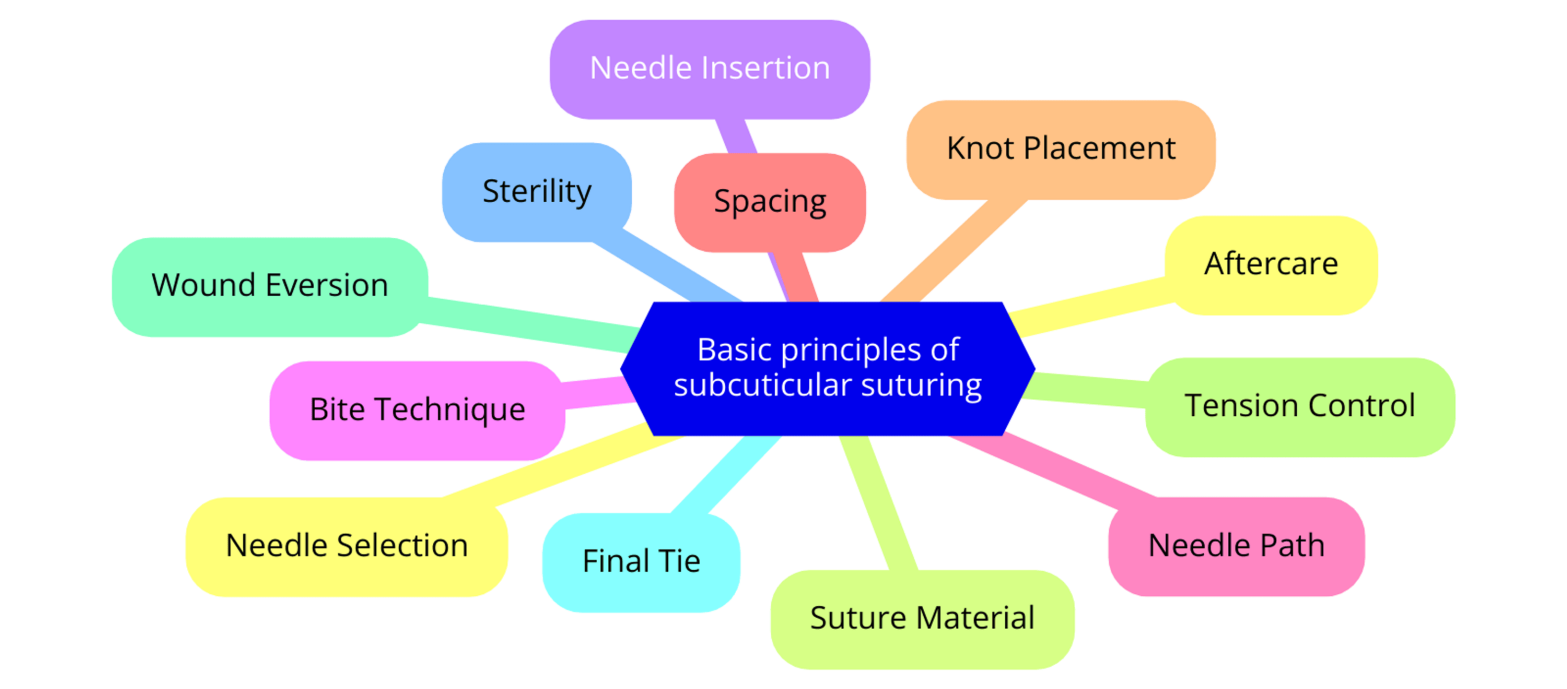
Basic principles of subcuticular suturing Image credit: Maulik Maheshwari

Subcuticular suturing is a technique employed to close wounds by placing sutures beneath the epidermis, thereby minimizing scarring and enhancing aesthetic outcomes. The primary principle involves suturing beneath the epidermis to reduce the number of puncture points, thereby lowering the risk of suture-track scarring and improving cosmetic results [7]. Another critical principle is using absorbable suture material, typically preferred to mitigate infection and foreign body reactions. While nonabsorbable sutures can be utilized, monofilament suture material is recommended to decrease the friction coefficient during removal [8]. Various subcuticular suturing techniques exist, including the running subcuticular, interrupted subcuticular, and buried vertical mattress sutures, each offering distinct advantages suitable for different wound types and locations. However, these sutures are best applied to wounds under minimal to no tension and should be avoided in areas with thin dermis or atrophic skin [5]. Selecting appropriate instruments is crucial for effective subcuticular suturing. Curved needles are commonly used, and a palm grip on the holder allows optimal wrist mobility. Knots should be tied deeply at the subcutaneous level to minimize suture protrusion and infection risk; knot-free techniques can also be employed to mitigate these issues [9]. The timing for suture removal varies based on wound location and closure tension. Typically, sutures on the head and neck are removed five to seven days postoperation, whereas those on the trunk or extremities are removed between 10 and 14 days. These principles ensure that subcuticular suturing achieves optimal wound closure with minimal scarring and improved aesthetic results [10]. Basic principles of subcuticular suturing are shown in Figure 1.

Various Techniques and Methods 

Subcuticular suturing is extensively employed for closing abdominal wall incisions following laparotomy procedures. The continuous subcuticular suture technique, utilizing slowly absorbable monofilament suture material, has demonstrated superiority over interrupted sutures in reducing the incidence of incisional hernias. Studies indicate that maintaining a suture length to wound length ratio of at least 4:1 significantly decreases the risk of developing such hernias compared to interrupted sutures [11]. A notable advancement in subcuticular suturing is the adoption of the "small-bite" technique, where sutures are placed closer together (4-5 mm apart) and nearer to the wound edge (5-8 mm). This method has proven more effective than the "large-bite" technique by distributing tension more evenly across the wound, thereby lowering the risk of "button-hole" hernias [12]. The selection of suture material is critical in subcuticular suturing. Slowly absorbable monofilament sutures such as polydioxanone (PDS) or poliglecaprone 25 (Monocryl) are preferred over rapidly absorbable or braided sutures. These materials offer superior long-term strength and support for healing the abdominal wall [13]. For patients at high risk of incisional hernia, prophylactic mesh reinforcement during midline laparotomy closure has shown significant benefits compared to suture closure alone. This approach is particularly recommended for patients with obesity, prior hernias, or other predisposing factors [14].

Advances in subcuticular suturing

Technological Innovations

One significant technological advancement in suturing is the Suture Tool, a mechanical needle driver designed to enhance standardized wound closure, particularly in abdominal wall procedures. This tool features a double-pointed needle with a centrally attached thread, a guiding mechanism for precise stitch placement and jaws that compress to pass the needle through tissue, automatically picking it up on the opposite side [15]. The Suture Tool aims to achieve a suture length-to-wound length (SL/WL) ratio of ≥4:1, critical for reducing the risk of incisional hernias. In comparative studies with conventional needle driver suturing (NDS), the Suture Tool achieved a ≥4:1 SL/WL ratio in 95% of cases, compared to only 30% with NDS. Moreover, using the Suture Tool reduced suturing time by 30%, highlighting its efficiency and potential to enhance surgical outcomes [16]. Another notable advancement in suturing technology involves using slowly absorbable monofilament sutures like polydioxanone (PDS) and poliglecaprone 25 (Monocryl). These materials offer several advantages over traditional options. Their slow absorption rate provides prolonged support and strength for the healing abdominal wall, while their monofilament structure reduces infection risks and other complications associated with braided sutures. Studies have consistently demonstrated the superior performance of these advanced suture materials regarding strength retention and tissue compatibility, making them preferable for abdominal wall closure procedures [17].

Enhanced Techniques

Advanced techniques in subcuticular suturing have transformed surgical practices by offering innovative alternatives to traditional methods. One notable advancement is knotless suturing, eliminating the need for knots, thereby reducing complications such as knot slippage, extrusion, and infection risks. This technique employs sutures with barbs or other anchoring mechanisms that securely hold the suture without traditional knots. Knotless suturing has been widely adopted across various surgical disciplines, including cosmetic, urological, general, orthopedic, obstetric, and gynecological surgeries [18]. Another significant enhancement is barbed sutures, which feature barbs along their length to anchor securely in tissue without knots. These sutures come in bidirectional and unidirectional types, such as the Quill SRS, V-Loc absorbable wound closure device, and Stratafix. While barbed sutures can reduce suture and operative times compared to conventional sutures, they may also pose a higher risk of postoperative complications [19]. Tissue adhesives represent another advanced technique for wound closure, offering a suture-free option. These adhesives are applied directly to the wound site to seal it shut and promote healing by bonding the wound edges. They are particularly useful in minor surgical procedures where suturing may be challenging or impractical, such as skin closures. Tissue adhesives improve safety and efficiency in wound closure procedures, especially when sutures are not ideal [20].

Clinical outcomes and efficacy

Healing and Cosmesis

According to studies, the cosmetic outcomes of subcuticular suturing for abdominal wall closure generally compare favorably with other closure techniques, such as skin staples and tissue adhesives. Research indicates that there is typically no significant difference in scar assessment scores between subcuticular sutures and alternative methods [21]. However, nuances exist among different suturing techniques. For instance, one study highlighted that the intradermal buried vertical mattress suture technique could yield superior cosmetic outcomes to the traditional subcuticular suture method, particularly in cesarean section scars [22]. This suggests that the specific suturing technique can impact the final aesthetic result. Regarding patient satisfaction, subcuticular sutures may offer a slight advantage over skin staples in the short term (within 30 days post-procedure). However, patients may express slightly less satisfaction with subcuticular sutures than tissue adhesives during the initial 14-21 days after surgery [21]. One trade-off associated with subcuticular suturing is the increased time required for closure. Studies have demonstrated that subcuticular suturing generally takes a few minutes longer than skin stapling or tissue adhesives. This aspect is particularly important in surgical settings where time efficiency is critical [21]. While subcuticular suturing can achieve good cosmetic outcomes, its advantage over other closure methods appears modest. Proper selection of suturing technique and suture material are crucial in optimizing healing and cosmesis. Surgeons should carefully consider the minor differences in cosmesis, patient satisfaction, and closure time when deciding on the most appropriate closure method for individual patients [21].

Infection Rates and Complications

The evidence regarding whether subcuticular sutures or skin staples offer a lower risk of surgical site infections (SSIs) for abdominal wall closure is varied. While one meta-analysis found no significant difference in SSI rates between subcuticular sutures and staples for abdominal surgeries, individual studies have suggested potential advantages of subcuticular sutures, especially in clean surgical procedures such as cesarean sections [23]. The impact of closure technique on SSI risk may depend on factors specific to the surgical site and procedure. For instance, research has shown no difference in SSI rates between sutures and staples in craniofacial surgery [23]. Evidence in cases involving contaminated wounds shows that using buried absorbable subcutaneous sutures (BASS) may increase the risk of infection compared to transdermal sutures without subcutaneous sutures. This increased risk is observed even after thorough irrigation, likely due to the presence of buried suture material contributing to microbial colonization [24]. While subcuticular suturing may take slightly longer than staples or tissue adhesives, this does not typically significantly impact outcomes such as wound dehiscence or cosmetic results. Patient satisfaction with subcuticular sutures may be marginally higher in the short term than staples but generally lower compared to tissue adhesives [25]. The choice between subcuticular sutures, skin staples, or other closure methods should consider the specific clinical context, including the type of surgery, wound contamination risk, and patient preferences. Each closure technique has its own advantages and potential risks, which surgeons should carefully weigh to optimize surgical outcomes and patient satisfaction.

Patient Satisfaction and Pain Management

Subcuticular suturing is widely favored for abdominal wall closure following laparotomy procedures due to its effectiveness in reducing the incidence of incisional hernias. The continuous suture technique using slowly absorbable materials such as polydioxanone (PDS) or poliglecaprone 25 (Monocryl) has been particularly successful. Studies indicate that maintaining a SL/WL ratio of at least 4:1 significantly decreases the risk of incisional hernias compared to interrupted sutures. Additionally, adopting the "small-bite" suturing technique, where sutures are placed closer together (4-5 mm apart) and nearer to the wound edge (5-8 mm), helps distribute tension evenly across the wound, further reducing the risk of complications like "button-hole" hernias [11]. The choice of suture material plays a crucial role, with slowly absorbable monofilament sutures preferred over rapidly absorbable or braided options due to their superior long-term strength and support for healing the abdominal wall. For patients at high risk, prophylactic mesh reinforcement during midline laparotomy closure has been shown to significantly lower the incidence of incisional hernias compared to sutures alone, especially beneficial for individuals with obesity or prior hernias [13]. The Suture Tool represents a technological advance in wound closure, designed to enhance the quality and efficiency of suturing in abdominal wall procedures. This mechanical needle driver ensures a high-quality suture line by achieving an SL/WL ratio of ≥4:1 in most cases, compared to conventional needle driver suturing methods. It has been demonstrated to reduce suturing time by 30%, underscoring its potential to improve surgical outcomes [15]. While subcuticular suturing offers advantages such as lower incisional hernia rates and favorable cosmetic outcomes, it may require more time than alternatives like staples or tissue adhesives. Patient satisfaction with subcuticular sutures can be high, particularly in the short term, although closure time considerations should be weighed in clinical decision-making. Overall, subcuticular suturing is recognized for its efficacy in achieving optimal surgical outcomes and patient satisfaction in abdominal wall closure procedures.

Cost-Effectiveness and Resource Utilization

Subcuticular suturing is a widely adopted technique for closing abdominal wall incisions following laparotomy procedures, primarily due to its effectiveness in reducing the risk of incisional hernias. The continuous suture technique using slowly absorbable suture materials like polydioxanone (PDS) or poliglecaprone 25 (Monocryl) has successfully achieved this outcome. Research indicates that maintaining an SL/WL ratio of at least 4:1 significantly lowers the incidence of incisional hernias compared to interrupted sutures [11]. In addition to the continuous suture technique, the "small-bite" suturing approach has been shown to enhance outcomes by placing sutures closer together (4-5 mm apart) and nearer to the wound edge (5-8 mm). This method effectively distributes tension evenly across the wound, reducing the risk of complications such as "button-hole" hernias [11]. The choice of suture material is critical in subcuticular suturing. Slowly absorbable monofilament sutures are preferred over rapidly absorbable or braided options due to their superior long-term strength and support for healing the abdominal wall [26]. This choice is pivotal in optimizing wound closure and minimizing complications. For patients at elevated risk, such as those with obesity, previous hernias, or other predisposing factors, the use of prophylactic mesh reinforcement during midline laparotomy closure has been shown to significantly decrease the likelihood of developing incisional hernias compared to relying solely on sutures. This approach is recommended to enhance the structural integrity of the abdominal wall postsurgery and mitigate the risk of herniation [27].

Case Studies and Clinical Trials

Advances in suturing devices and techniques have significantly enhanced abdominal wall closure procedures, focusing on improving both efficiency and outcomes. The Suture Tool, a mechanical needle driver designed for standardized wound closure, has substantially improved over conventional methods. Studies have shown that the Suture Tool achieves a high SL/WL ratio of ≥4 in 95% of cases, compared to only 30% with traditional needle drivers. Moreover, it reduces suturing time by 30%, highlighting its ability to streamline the closure process and potentially improve surgical efficiency [15]. Clinical trials and systematic reviews have underscored the benefits of specific suturing techniques and materials in reducing complications such as incisional hernias. A comprehensive review involving over 7,400 participants found that the continuous suture technique using slowly absorbable materials significantly lowers the incidence of incisional hernias compared to interrupted sutures, particularly when maintaining an SL/WL ratio of at least 4:1 [28]. Similarly, studies comparing suturing techniques, such as the "small-bite" method versus "large bites," have shown that closer suture placement reduces tension across the wound, thereby decreasing the risk of complications like "button-hole" defects and subsequent hernias [29]. For patients at high risk of incisional hernias, such as those with obesity or prior hernias, prophylactic mesh reinforcement during midline laparotomy closure has emerged as an effective strategy. Clinical trials have demonstrated that placing a lightweight, macroporous synthetic mesh to bolster the fascial closure significantly reduces hernia incidence compared to suturing alone [30]. This approach enhances the structural integrity of the abdominal wall postsurgery, thereby mitigating the risk of herniation. Meta-analyses have evaluated different closure techniques for SSIs. One meta-analysis involving over 3,700 patients undergoing abdominal surgery found no significant difference in SSI rates between subcuticular sutures and skin staples overall. However, in specific subgroups such as gastrointestinal surgery, subcuticular suturing showed comparable SSI risks to skin stapling, highlighting the importance of tailored closure methods based on surgical context and patient factors [31].

Challenges and limitations

Subcuticular suturing represents an advanced closure technique that offers potential cosmetic benefits but requires a more substantial learning curve than traditional interrupted suturing methods. Mastery of subcuticular suturing demands advanced technical skills and experience from surgeons to achieve optimal outcomes and mitigate potential complications [5]. Inexperienced surgeons may encounter challenges such as suture breakage, wound dehiscence, and suboptimal cosmetic results when subcuticular closures. These complications can arise due to the intricate nature of the technique, which involves placing sutures beneath the epidermis to minimize visible scarring and enhance aesthetic outcomes [5]. Patient-specific factors also play a crucial role in determining the suitability of subcuticular suturing. Thin or fragile skin poses a higher risk of suture extrusion through the epidermis, compromising the closure's effectiveness and potentially leading to complications. Patients predisposed to hypertrophic scarring may not fully benefit from the cosmetic advantages of subcuticular suturing, as this technique does not alter the underlying propensity for excessive scar tissue formation [5]. Anatomical considerations further influence the applicability of subcuticular closure. High-tension areas or anatomical sites prone to movement and stress may pose challenges for achieving durable closure solely through subcuticular suturing. In such cases, adjunctive techniques like mesh augmentation may be necessary to reinforce the abdominal wall or other anatomical structures [5]. While subcuticular suturing can yield favorable short-term cosmetic outcomes, the long-term results can vary depending on suture material and technique. Absorbable sutures like polydioxanone (PDS) and poliglecaprone 25 (Monocryl) are often preferred over nonabsorbable options for their reduced risk of long-term complications such as suture extrusion or granuloma formation [21]. Continuous subcuticular sutures have been shown to lower the risk of superficial wound dehiscence, underscoring their utility in promoting wound healing and minimizing postoperative complications. However, selecting the optimal suture type and technique remains contingent on the specific surgical context, including the nature of the procedure and the patient's characteristics [21].

Future directions

The future of subcuticular suturing for abdominal wall closure is poised for exciting advancements and innovations across several key areas. One promising avenue of development lies in automated suturing devices designed to standardize the technique and enhance efficiency. Devices like the Suture Tool, a mechanical needle driver, exemplify this trend by ensuring precise suture placement and maintaining a consistent suture length-to-incision length ratio. By reducing the variability inherent in manual suturing, these devices have the potential to streamline and improve the quality of abdominal closure procedures [15]. Simultaneously, ongoing research focuses on optimizing suture materials to further enhance their performance characteristics. While slowly absorbable monofilament sutures such as polydioxanone (PDS) and poliglecaprone 25 (Monocryl) have shown considerable benefits in terms of strength, absorption rates, and tissue compatibility, continuous advancements are being pursued to refine these properties [32]. The expanded use of prophylactic mesh reinforcement during midline laparotomy closure represents another promising direction. This technique has demonstrated substantial reductions in incisional hernias, which is particularly beneficial for patients at higher risk due to factors like obesity or previous hernias [33]. As minimally invasive surgical techniques become increasingly prevalent, adapting subcuticular suturing techniques for laparoscopic and robotic-assisted procedures is becoming crucial. Specialized suturing tools and techniques tailored to these platforms are being developed to address challenges such as the longer learning curve and increased intraoperative time associated with these approaches compared to traditional open surgeries [33]. Looking ahead, personalized suturing strategies based on individual patient risk factors are also emerging as a potential future trend. Tailoring techniques such as small bites, using specific suture materials, and selecting a prophylactic mesh for high-risk patients can optimize outcomes and minimize complications like incisional hernias. However, careful consideration of patient-specific factors, such as skin condition and surgical history, is essential to ensure the suitability and effectiveness of these personalized approaches [34].

## Conclusions

In conclusion, subcuticular suturing for abdominal wall closure has proven an effective technique that offers significant advantages in cosmetic outcomes, reduced infection rates, and patient satisfaction. The evolution from traditional suturing methods to advanced techniques, such as barbed sutures and tissue adhesives, reflects the ongoing commitment to improving surgical outcomes and patient care. Despite the challenges and technical demands associated with subcuticular suturing, its benefits make it a valuable option in various surgical specialties. Continued research and innovation in this field promise further enhancements in technique and materials, potentially leading to more efficient and effective closure methods. This review underscores the importance of integrating new advancements with established practices to optimize abdominal wall closure, ultimately contributing to better patient outcomes and advancing the field of surgery.
